# Psychologists’ experiences with telepsychology: qualitative analysis employing GDEISST framework

**DOI:** 10.3389/fdgth.2025.1621551

**Published:** 2025-10-03

**Authors:** Tamadhir Al-Mahrouqi, Zena M. Al-Sharbati, Kamila Al-Alawi, Ahmed AlHarthi, Asayel Al Siyabi, Mohammed Al-Alawi, Shaima Al Humimia, Muna Al Salmi, Tharaya Al-Hashemi, Rahma Al Nuumani, Fatma Al Balushi, Hamed Al Sinawi

**Affiliations:** ^1^ Department of Behavioral Medicine, College of Medicine and Health Sciences, Sultan Qaboos University, Muscat, Oman; ^2^ Sultan Qaboos Comprehensive Cancer Care and Research Center, Muscat, Oman; ^3^ Oman Medical Specialty Board, Muscat, Oman; ^4^ Al Masarra Hospital, Ministry of Health, Muscat, Oman

**Keywords:** telepsychology, telemedicine, teletherapy, telehealth, Oman

## Abstract

**Objective:**

To explore Omani psychologists’ perceptions and experiences of telephone-based psychotherapy consultations, specifically regarding service quality, accessibility, and acceptability.

**Methods:**

An exploratory qualitative study was conducted at Al Masarra Hospital in Muscat, Oman, between December 1 and 31, 2024. Five psychologists who provided telephone-based consultations were purposively sampled and participated in semi-structured, face-to-face interviews guided by the first three domains of the GDEISST framework (quality of services, accessibility, and acceptability). The acronym GDEISST stands for the “Guide for the Design, Evaluation, and Implementation of Telemedicine-Based Health Services.” Data analysis was conducted using inductive thematic analysis, while being guided by the first three domains of the GDEISST framework.

**Results:**

Telephone-based psychotherapy quality is optimized by adopting a hybrid model after in-person sessions to establish trust and using clear, intentional communication supported by formal guidelines and training to compensate for lost non-verbal cues. Accessibility is hindered by client-side factors (privacy, scheduling, connectivity) and institutional constraints (limited phone lines, private spaces, and trained personnel). Finally, telephone consultations are broadly acceptable, particularly for clinically stable or stigma-sensitive clients, who appreciate the convenience and discretion they offer, but are considered unsuitable for high-risk cases such as active psychosis or suicidal ideation.

**Conclusion:**

Telephone-based psychotherapy is viewed by Omani psychologists as a valuable adjunct to traditional care, enhancing access, reducing stigma, and supporting maintenance of stable clients provided that hybrid models (initial face-to-face engagement), structured training, and robust infrastructure are in place. However, high-risk or acutely unwell clients require in-person evaluation to ensure safety and treatment efficacy.

## Introduction

Telepsychology, which involves delivering psychological services remotely via telecommunications technology, has become a revolutionary method in global mental health care, especially after the COVID-19 pandemic (1). The pandemic caused significant healthcare delivery disruptions, reducing non-essential services and in-person appointments to safeguard patients and medical personnel. This disruption could have exacerbated illnesses and intensified anxiety, ultimately negatively affecting patients’ overall quality of life (2). Therefore, governments and healthcare systems globally have reorganized mental health services to maintain accessibility (3, 4). This progressive practice utilizes video conferencing, telephone consultations, and secured messaging to connect mental health providers with clients across different geographical locations while encouraging physical distancing (5).

Telepsychology is not a novel practice, rather, it is a healthcare service delivery method that was less commonly utilized before the COVID-19 pandemic (1). From 2000 to 2008, the use of telepsychology technology among psychologists in government institutions of the United States of America rose from 2% to 10%. In addition, there was a dramatic increase in its utilization during the pandemic. In the latest surveys, approximately 40% of psychologists and psychiatrists who use telepsychology in the U.S. reported safe utilizing videoconferencing technology (VCT) as a complementary approach to face-to-face services, while nearly 45% of them indicated that they used VCT as a standalone method (6).

Telepsychology provides numerous advantages to the clients receiving this service. More specifically, it connects distant locations and offers essential access to mental health specialists in rural areas while saving time and travel expenses (3). In addition, telepsychology facilitates early interventions and enhances collaboration among various healthcare providers simultaneously through a streamlined process (7). Many studies indicated that telepsychology enhances client-reported outcomes. For example, a meta-analysis study demonstrated that telepsychology interventions significantly improved symptoms across diverse client populations suffering from depression, anxiety disorders, and post-traumatic stress disorder (PTSD) (8). Furthermore, telepsychology is linked to elevated levels of client satisfaction, as many clients express feeling more at ease with less self-stigma when receiving mental health services remotely compared to traditional in-person sessions. This increased comfort often results in improved therapy engagement and greater adherence to treatment plans, ultimately resulting in improved long-term client-reported outcomes (9).

Telephone consultations are widely used in global telehealth, particularly in primary care in the United Kingdom and internal medicine in the U.S.A. Evaluating physicians’ and clients’ satisfaction is crucial to understand the benefits, drawbacks, and areas requiring improvement in these consultations (4).

Telephone consultations are essential in telepsychology, particularly in areas with unreliable internet access. They provide a dependable means to deliver mental health services remotely, ensuring consistent care despite technological limitations. Additionally, clients often prefer telephone consultations due to their convenience and enhanced privacy compared to video calls or face-to-face visits. This preference frequently leads to increased therapy engagement and better adherence to treatment plans, underscoring the role of telephone consultations in improving access to mental health care (10).

On the other hand, telephone consultations have their drawbacks as well. For instance, they often prioritize addressing immediate symptoms, potentially resulting in a less comprehensive approach to the clinical psychiatric/psychological evaluation of clients. The absence of visual and non-verbal cues, such as body language and facial expressions, and the inability to conduct physical examinations are additional limitations of telehealth. Furthermore, challenges include issues related to maintaining effective physician-client relationships and navigating the bureaucratic complexities that could exist (11).

A survey study indicated that psychotherapists perceive telepsychology sessions as less emotionally profound compared to in-person sessions, often leading to a sense of emotional disconnection from their clients (12). In these sessions, psychotherapists tend to focus more on support and counselling rather than a deeper exploration of emotions, often adopting a more active and directive approach (12). Additionally, psychotherapists reported some difficulties in maintaining their focus and presence during telepsychology compared to in-person sessions, which could influence the therapeutic alliance (12). Another qualitative study examining psychiatrists’ and psychologists’ experiences in tele-mental health revealed that participants valued teamwork. However, participants reported challenges in communication with primary health-care providers, particularly within the telehealth referral model, including symptom monitoring, scheduling, electronic medical record management, and credentialing (13). In Oman, telehealth was unavailable before the COVID-19 pandemic (2). During the pandemic, a randomized clinical trial conducted in Oman found that therapist-guided online therapy for the treatment of symptoms of anxiety and depression yielded substantial improvements in reducing clients’ symptoms in comparison to a self-help email group (2). Additionally, another study explored the Omani psychiatrists’ perceptions towards telephone consultations, where they revealed communication constraints and infrastructure problems related to telephone consultations. Despite the expected challenges, telephone appointments were perceived by psychiatrists as a convenient option to enhance clients’ adherence and accessibility to mental health services (5). Assessing and addressing these challenges is crucial for enhancing telepsychology services in Oman. By tackling these challenges, we could ensure that more people would have access to mental health services. This study fills a void in the current literature by focusing on Omani psychologists’ perceptions of telephone-based telepsychology consultations in Oman, specifically from three aspects: quality, accessibility, and acceptability. Al Masarra Hospital (AMH) is the only tertiary psychiatric hospital in Oman and currently hosts the country's only psychology department offering telepsychology services. This field of study has not been previously explored from the psychologists’ perspective; prior research has focused solely on psychiatrists’ viewpoints. This qualitative exploration will be the first of its kind in Oman and the Middle East, utilizing semi-structured interviews to gain a deep understanding of psychologists’ perceptions and viewpoints on telepsychology. The research outcomes are anticipated to make a significant theoretical and practical impact on the service by expanding our understanding of telepsychology in Oman, thereby informing policymakers, training entities, and infrastructure advisors about improvements to enhance mental health care services in the area.

## Methods

### Study design

Given the scarcity of literature on the effectiveness, quality, safety, and use of telephone consultations as a service provided by psychologists in the Middle East, this research utilizes an explorative qualitative approach. This study employed individual semi-structured face-to-face interviews to evaluate the psychologists’ perspectives on the quality, accessibility, and acceptability of telepsychology services in Oman.

Although there have been no official efforts in the country to evaluate telepsychology services, less attention has been paid to evaluating the quality of care, accessibility, and acceptability of mental health services. This study adopts the (GDEISST) framework (14) to evaluate telephone-based psychotherapy service as reflected by the psychologists’ broad experiences.

The (GDEISST) in Spanish stands for “Guía de diseño, evaluación e implantación de servicios de salud basados en telemedicina” authored by Serrano and Yanes, and published in 2006 (14). It is translated in English as: “Guide for the Design, Evaluation, and Implementation of Telemedicine-Based Health Services”. The GDEISST framework presents a comprehensive social approach to the evaluation of telemedicine. The GDEISST conceptual framework consists of five domains: Evaluation of the quality of telemedicine services, Evaluation of access to telemedicine services, Evaluation of acceptability of telemedicine services, Evaluation of the impact of telemedicine costs, and Evaluation in the healthcare organization. This study focused on the analysis of the first three domains (14). These were selected because they are the most directly relevant to patient-centered care and user experience, which are critical in understanding the feasibility and effectiveness of telephone-based telepsychology from the psychologists’ perspective.

### Study setting

All interviews were conducted at Al Masarra Hospital (AMH) in private rooms at the psychologists’ convenient timings. The AMH was established in 2013 in Muscat and is the only psychiatric hospital in Oman. It offers comprehensive mental health services, including General Adult Psychiatry, Geriatric Psychiatry, Child and Adolescent Psychiatry, and Forensic Psychiatry. Additionally, the hospital provides treatment for alcohol and substance misuse, along with services related to psychology, social work, physiotherapy, occupational therapy, and speech therapy.

### Participants

A total of seven psychologists working at AMH were approached in person and invited to participate in the study. The participants received an electronic consent form, including the time and place of the meeting, via their institutional emails. The aim and objectives of this study, alongside the benefits of the psychologist's participation, were explained in person, with the reassurance of maintaining confidentiality throughout the preparation process of this study.

### Inclusion and exclusion criteria of study participants

All psychologists who participated in telephone consultations at AMH and voluntarily submitted the electronic consent form were eligible to participate in this study.

### Data collection

Data collection took place between the 1st and 31st of December 2024, in which purposive sampling was applied. Five out of seven psychologists agreed to participate in the study. All participants in this study are licensed psychologists who provide psychotherapy, including evidence-based approaches such as Cognitive Behavioral Therapy (CBT). The participants’ information is presented in Table 1. Data collection was stopped when saturation was reached (15). All interviews were conducted by the first author, a female Omani senior psychiatry resident, with no prior relationships established with the participants. The interview guide was constructed in Arabic and English with open-ended and closed-ended questions related to telepsychology consultations (Box 1). The average duration of each interview was approximately 30 min, and to ensure high ethical conduct, all interviews were audio-recorded, transcribed verbatim, translated to English when necessary and stored securely.

**Table 1:** Participants charasteristics.

Participant	Gender	Unit	Years of experience in speciality	Years of experience with tele-pscyhology	Previous training in tele-psychology	Therapeutic orientation	Target symptoms	Target population
1	Female	General adult unit	7 years	3 years	No	CBT	Anxiety, depression, bipolar symptoms, psychosis, Trauma, substance abuse, adjustment disorders, stress, cultural issues	Adults, adolescents, women
2	Female	General adult unit	15 years	4 years	No	Psychodynamic, Gestalt,	Anxiety, depression, trauma, stress, cultural issues	Adults
3	Female	General adult unit	11 years	4–5 years	Yes	CBT	Anxiety, Depression, Bipolar symptoms, Trauma, Grief, Adjustment disorders, Stress	Adults
4	Female	Child and Adolescent unit	10 years	2 years	No	CBT	Anxiety, depression, adjustment disorders, Stress	Children, adolescents
5	Female	General adult unit	11 years	5 years	No	CBT, DBT, general Life-skills	Anxiety, depression, bipolar symptoms, substance abuse, life transition, grief, bereavement, adjustment disorders, stress, cultural issues	Adults, couples, women

Box 1 **Semi-structured Interview Guide:**

1. Can you share your experiences with providing telephone-based psychotherapy?
  1.1 Prompt: how has this modality impacted your practice?
2. What do you think is necessary to establish a strong therapeutic relationship in telephone-based therapy consultations, while ensuring the client's safety and maintaining ethical treatment?
3. Do you believe telephone-based therapy can effectively provide therapeutic relief?
4. Based on your experience, what forms of therapy have you provided to clients over the phone? (e.g., CBT, ACT, supportive therapy)
5. What concerns have you experienced when providing telephone-based therapy consultations?
6. From your experience, what concerns do clients typically have about receiving telephone-based therapy?
7. In your view, under what conditions would telephone-based therapy be considered an inappropriate model for managing a clients?
8. Are there instances in which clients might prefer telephone-based therapy therapy over face-to-face therapy?
9. Do you feel that your education and training adequately prepared you for conducting therapy sessions over the phone?
  9.1 Prompt: Why or why not?

### Data analysis

The interviews were analyzed primarily using an inductive approach while acknowledging the first three domains of the GDEISST framework. The interviews were transcribed verbatim without the use of transcription software. All transcripts were manually prepared, allowing for the careful identification of codes relevant: Evaluation of the quality of telemedicine services, evaluation of access to telemedicine services, and evaluation of acceptability of telemedicine services. Data analysis was initiated after the first interview, and to ensure a common understanding of the transcribed data, three researchers were involved in the analysis. Two of them, TM and ZS, are team members who independently reviewed the transcripts to identify the superordinate and subordinate themes. Then, KA, as an external researcher, reviewed the themes, and collectively, all researchers agreed on the final themes.

### Ethical considerations

Ethical approval was obtained from the Research and Ethics Committee at the Ministry of Health, Oman, with approval number (MoH/CSR/24/29102). All participating psychologists provided written informed consent before their involvement in the research. Participants were thoroughly informed about the study's objectives, procedures, and their rights, including the voluntary nature of their participation and freedom to withdraw from the study at any time without any negative consequences. Confidentiality and anonymity were rigorously maintained throughout the research process, ensuring that all data were securely stored and accessible only to the research team.

## Results

The results will be presented under three domains: Evaluation of the quality of telemedicine services, evaluation of access to telemedicine services and evaluation of acceptability of telemedicine services, which will be referred to as three superordinate themes. Then, the three superordinate themes will be further divided into several subordinate themes as described in Table 2.

**Table 2:** Translating domains into themes of analysis.

Superordinate themes	Subordinate themes
Evaluation of the quality of telemedicine services.	1. Conditions and their associated factors that ensure high-quality telemedicine sessions. 2. Challenges that may hinder quality of telemedicine sessions.
Evaluation of accessability to telemedicine services.	1.Client-related contextual factors that may hinder access to telemedicine sessions. 2. Psychologist-related factors that may affect accessability to telemedicine sessions.
Evaluation of acceptability of telemedicine services.	1. Clients acceptability of telemedicine. 2. Conditions and their associated factors that may affect acceptability of telemedicine sessions.

The first superordinate theme discusses the conditions, from the psychologists’ point of view, that help to establish high-quality telemedicine sessions. Additionally, it debates the challenges of this type of service within the unique Omani cultural context. The second superordinate theme argues the accessibility of telemedicine sessions, reflecting accessibility issues from the psychologists’ and clients’ perspectives. Finally, the third superordinate theme reviews clients’ acceptability of the service and discusses certain clinical conditions that make it unacceptable to provide telemedicine services

### Evaluation of the quality of telemedicine services

The psychologists emphasized the importance of meeting certain standard requirements for high-quality telepsychology sessions. They emphasized that an initial in-person session is essential for building rapport and observing body language, which are key elements in understanding the client. They also suggested that ideally, five in-person sessions should take place before transitioning to telepsychology, ensuring that a strong therapeutic relationship is established. This reflects a hybrid approach, where in-person interaction lays the foundation for effective remote care, addressing concerns regarding the limitations of virtual therapy in fostering early engagement and trust.

“An initial in-person session is crucial for establishing rapport and understanding body language. Ideally, five in-person sessions would be beneficial before transitioning to telepsychology. In my view, tele-therapy should only begin once a solid relationship is established.”

The psychologists acknowledged that telephone-based psychotherapy poses unique challenges, most importantly, the need to build therapeutic trust through several initial face-to-face sessions to optimize the quality of subsequent remote encounters is essential. They further emphasized that a strong, pre-existing therapeutic alliance is vital for sustaining the effectiveness and overall success of telepsychology.

“The most important factor is the client’s trust in the confidentiality of the call. If they trust the psychologist and the privacy of the conversation, they are more likely to continue the sessions, which contributes to strengthening the therapeutic alliance. The initial in-person session forms the foundation for building trust between the psychologist and the client. If trust isn’t established during the first session, we invite the client for another in-person session to reinforce and build that trust before deciding whether to proceed with an in-person or telephone session.”

The psychologists at the hospital developed guidelines to further improve the quality of telepsychology sessions.

“We developed new guidelines specifically for telephone-based psychotherapy.”

The psychologists discussed clients’ personal growth through unique communication skills during telepsychology. By learning to use clearer language, asking more targeted questions, and adopting an affirmative tone, the therapist could enhance client understanding and engagement. This adaptation underscores the importance of intentional communication in remote settings, where non-verbal cues are limited and clarity becomes crucial for effective care.

“I learned to use clearer language, ask more specific questions, and adopt an affirmative communication style to improve understanding and address patients’ concerns effectively”.

Even though these psychologists appreciated the potential conditions that could enhance the quality of the telemedicine session, they also reflected upon some challenges of this type of service. In the following excerpts, the subordinate theme (*Challenges that may hinder the quality of telemedicine*) will be discussed.

Face-to-face therapy is often preferred over telepsychology sessions and deemed more effective because it enables richer nonverbal communication, such as body language and facial expressions, fostering deeper engagement and helping therapists in detecting subtle cues that enhance treatment effectiveness.

“Face-to-face therapy is preferred for greater effectiveness, as it allows for better non-verbal communication and deeper engagement”.

Accurately assessing issues such as medication adherence or hallucinatory experiences is more difficult through a phone consultation, especially with new clients who may conceal or underreport symptoms. While long-term clients often reveal inconsistencies over time, psychologists must remain vigilant and consider supplemental strategies such as in-person visits or receiving collateral information from family members and caregivers to ensure an accurate understanding of the client’s condition.

“Assessment accuracy is essential, it’s harder to assess issues like medication adherence or hallucinatory behaviours over the phone. Inconsistencies can be easily detected with long-term patients, but the challenge is with new patients who might hide symptoms or fail to provide an accurate picture of their condition”.

“Also, It is harder to assess a patient’s mood and affect accurately through phone calls”.

“Patients may not be honest or may minimise symptoms, especially if they lack privacy or feel uncomfortable disclosing personal information over the phone”.

Some psychologists have doubts about the effectiveness of conducting skill-based interventions remotely. Although techniques such as mindfulness exercises, breathing techniques, and progressive muscle relaxation are widely available online, they are perceived to be more effectively taught and practised in person, where real-time guidance and demonstration can enhance the learning experience.

“Although skills like mindfulness exercises, breathing techniques, and progressive muscle relaxation can be found on YouTube, they are more effectively demonstrated in person”.

“Efforts were made to send worksheets *via* email for telemedicine patients, but this proved impractical since many patients lacked email access or technical knowledge”.

Additionally, clients may try to shorten calls, which limits the therapist's ability to conduct thorough assessments. These behaviours reflect barriers to building trust and obtaining accurate clinical information in remote settings, potentially affecting the quality of care delivered.

“Some patients attempt to end calls quickly, making it harder to fully assess their symptoms”.

Another psychologist agreed with the above excerpt in that logistical issues and added that an appropriate space could be a challenge in conducting telemedicine sessions.

“Patients may also lack private spaces for phone calls, making them hesitant to share sensitive information”.

The participants mentioned additional challenges associated with delivering telepsychology sessions, which included formal education and training. Accordingly, they declared that they did not receive any formal training. Other psychologists mentioned that they involved themselves in self-learning activities, which helped them to engage in telepsychology.

“I did not receive formal training for telephone-based therapy. We just received a guide through a research study involving video-based therapy, and we followed a protocol for therapy sessions conducted *via* video conferencing during the COVID-19 pandemic. However, no specific training was provided for telephone-based therapy”.

Without ongoing training and professional development, psychologists may struggle to keep pace with evolving telepsychology methods. This gap can hinder their confidence and competence in delivering remote care, ultimately limiting the quality and effectiveness of telepsychology services.

“A lack of continuous support for training and professional development limits psychologists’ ability to stay updated with modern telepsychology techniques”.

### Evaluation of accessability to telemedicine services

Several challenges in accessibility to telepsychology were evident from various interviews conducted with psychologists. The most significant challenge was clients’ contextual factors, which negatively affected telepsychology sessions. For instance, telepsychology can be hindered by practical and environmental barriers that impede both access and quality of care. Many clients struggle to find a private space, especially during working hours or when family members are nearby, which inhibits open discussion and full engagement. Additionally, coordinating sessions around a client's limited availability complicates the scheduling process and increases the risk of missed appointments.

Even when contact is made, background noise or competing demands such as work and children can fragment concentration and derail the therapeutic process. Addressing these logistical challenges upfront by exploring alternative times, suggesting noise-reduction strategies, or identifying a more confidential setting ensures that telepsychology remains both accessible and effective.

“Patients may struggle to find a private space to speak freely, especially during working hours or when others are present. For example, I have been trying to contact a patient who can only answer at 10:00 am, which makes it challenging to schedule an appointment”.

“It can be difficult to reach some patients if they don’t answer the call or are occupied with work or family. Additionally, patients may become distracted by surrounding noise during the session”.

Unpredictable client engagement, such as missed calls, delayed responses, and the need for multiple rescheduling attempts, can impose a significant administrative burden on clinicians and disrupt the continuity of care.

“Some clients are unresponsive, missing calls, or requiring repeated attempts to schedule and reschedule appointments, which is time-consuming”.

Allowing clients to choose between in-person and telephone-based consultations addresses these accessibility challenges by accommodating individual preferences and logistical constraints. For clients who live far from the clinic or struggle to find consistent windows for appointments, the flexibility of telepsychology can reduce missed sessions and streamline scheduling. By offering both modalities, practitioners can minimize time spent on administrative follow-up, improve attendance rates, and ensure that care remains accessible and responsive to each client's circumstances.

“Patients are given the choice between in-person and telephone-based consultations, particularly if they live far away.”

In the child & adolescent age group, the psychologists reported that on many occasions, the consultations are limited to the parents since the consultations are made during the official working hours, and the children and adolescents are usually attending school and are not available to join the consultations.

“Communication often is limited to parents, as adolescents are unavailable, they are at school or lacking personal phones”

However, the challenge is not only from the client's side; the interviews shed light on psychologists’ related factors affecting accessibility to telemedicine services. The theme viewed the logistical as well as training challenges that this professional population faces regarding the service delivery of telemedicine. For instance, the psychologists shared that what makes access to telemedicine challenging in the first place is the general shortage of qualified psychologists at the national level, a lack of dedicated offices and landlines to enable safe telemedicine delivery, and a lack of subsidised training opportunities for psychologists.

“A shortage of psychologists at both the hospital and national levels adds significant pressure besides the limited resources available”.

“Some psychologists independently attended workshops and courses, but these are costly and not accessible to all”.

“A shortage of resources such as dedicated phone lines and private rooms for conducting telephone-based therapy could be a challenge”.

### Evaluation of the acceptability of telemedicine services

Several psychologists indicated that telemedicine has been exceptionally helpful with specific clients, including those who are psychologically stable and those with prior positive experiences in telemedicine. Others prefer telemedicine to avoid social encounters with people whom they may know as a consequence of social and self-stigma of mental illness.

Individuals with mental health concerns may avoid face-to-face consultations due to fear of social judgment, shame, or being labelled. In smaller communities, especially, where anonymity is limited, the risk of being seen entering a mental health facility can be a significant deterrent. As a result, tele-therapy emerges as a more discreet alternative, allowing clients to seek care in a way that preserves their privacy. Although the choice could not always be the most clinically preferred, it provides an essential bridge to care for those who might otherwise remain untreated due to stigma-related barriers.

“Others prefer tele-therapy due to the stigma associated with seeking a psychologist’s help. For example, some patients refuse to visit local centers because they fear being recognized, making telephone-based therapy the only viable option, even though it is not the ideal solution. However, it is better than no intervention at all.”

The psychologists pinpointed a consistent international problem of stigma against mental illness. This, in turn, would make telepsychology a helpful method to deliver mental health services to these clients. However, other clients might prefer telepsychology for different reasons, such as saving time and avoiding travel to the hospital, especially for those on supportive therapy.

“Clients might prefer telephone therapy if they want to save time, avoid long distances, or have transportation issues, if they have mild symptoms and require less frequent follow-ups with doctors, for example supportive therapy every 1–2 months as per their request”.

Integrating face-to-face sessions with subsequent telepsychology check-ins offers a powerful way to deepen the therapeutic alliance and keep adolescents actively engaged in treatment. An initial in-person meeting allows the therapist and teenager to establish rapport and build mutual trust foundations that carry over into telepsychology sessions. Follow-up via telepsychology not only sustains that connection between appointments but also provides timely prompts and accountability, reminding teenagers to complete homework assignments and practice new skills.

“Combining in-person sessions with telepsychology follow-ups enhances trust, maintains engagement and strengthens the therapeutic relationship, for teenagers, telepsychology services as a reminder to complete assigned tasks, encouraging them to take therapy seriously and improve compliance”.

Although clients generally find telemedicine acceptable, certain situations make its professional use inadvisable for psychologists. These include psychiatric emergencies with high risk to self or others, such as suicidal or homicidal ideation clients, clients with impaired insight, for example, those experiencing active psychosis or mania, and other severe psychiatric conditions, for example, clients with severe depression and panic attacks. Psychologists declared that these conditions must be identified and addressed before considering telepsychology, since clinical decisions in these contexts can have serious clinical and legal ramifications.

“Suicidal ideation, active psychosis, mania, severe depression, or panic attacks require in-person visits until the patients are stabilized. Patients in severe conditions are not suitable for telepsychology”.

“Patients with severe symptoms or suicidal thoughts often conceal the extent of their condition. Some explicitly insist on not informing their families of their suicidal ideation, placing the psychologist in a difficult position. In such cases, the psychologist advises the patient to seek emergency care and explains the importance of involving family members.”

## Discussion

Recently, telephone-based psychotherapy has become an essential method of service delivered to clients within the mental health care, especially post-COVID-19. Despite the importance and the significant demand for this service, evidence regarding its effectiveness and whether it is beneficial or suitable is underreported, particularly in the Middle East. This study explored the psychologists’ insights on telephone-based psychotherapy, focusing on three main domains: service quality, accessibility, and acceptability of telemedicine services. Results specified benefits and challenges faced during telephone-based psychotherapy while presenting methods to improve clients’ outcomes, alongside therapist satisfaction with developing a national framework for telephone-based psychotherapy.

Therapeutic alliance, an essential psychologist-client bond for effective clinical outcomes, is one of the most significant factors that can lead to successful telephone-based psychotherapy (16). Most participants agreed that an initial face-to-face visit is crucial to have a better telecommunication quality, in terms of building a good rapport and gaining clients’ trust, as well as ensuring that the client is receptive to the service. Also, according to the participants, patients’ body language and nonverbal communication are key in assessing mental health; however, restrictions may negatively impact the overall assessment of the client and weaken the quality of the service provided. Evidence showed that although in-person therapy sessions are valued for a better therapeutic alliance and their nuanced communication, a significant therapeutic alliance can be established using telephone-based psychotherapy despite the limitations in non-verbal signs (16). These results are relevant, particularly in societies where relational depth, collectivism, and trust are highly valued. While studies from Western settings have underlined the need for eye contact and non-verbal communication in therapy (3), our findings showed how strategic sequencing (e.g., beginning with face-to-face sessions) might help to overcome these restrictions. This adaptation indicated a grounded cultural attitude to preserving connection in the lack of visible signals. Furthermore, our results complemented earlier research by expanding therapeutic relationship models into understudied cultural settings such as Oman. In addition, technical difficulties like poor internet connection, availability of a suitable device, location, and the technical experience of both psychologists and clients could threaten the quality of the therapeutic process (17). Several studies agreed that the providers’ perception of telehealth is one of the significant factors affecting the quality and implementation of telephone sessions (3), in terms of concerns over the process of forming a good rapport, security concerns, technical issues, inappropriate settings, and infrastructure. However, following its implementation, they became more favourable, reporting easier access and service quality satisfaction (18).

This study is also aligned with another study (19) that indicated that telepsychiatry is mostly suitable for clients who are clinically and psychologically stable, and additionally helps in keeping them in remission.

The implementation of telephone-based psychotherapy in Oman has noticeable advantages, yet it is challenged by barriers that affect its accessibility and effectiveness. These barriers can be broadly categorized into geographical, technological, and sociocultural, which together affect the long-term success of such interventions. One of the shared responses was the ability of telepsychology to facilitate access to mental health services across Oman. Most participants stressed the significant time and financial resources that could be saved, especially with clients who have social, logistical, and financial difficulties, and these findings were also aligned with another study (20).

The lack of familiarity with telecommunications technologies among certain groups can lead to reluctance to engage in this form of therapy, particularly older populations that may prefer face-to-face interaction (21). One of the difficulties mentioned in our study was access to telepsychology sessions, and the difficulties associated with it. Some participants also reported hitches in arranging telephone sessions due to diverse client-psychologist scheduling. Therefore, time management was considered an essential tool during multiple attempts to reach the client. This could be overcome by having clear telemedicine scheduling guidelines that are shared between psychologists and their clients.

Insights from the current study highlighted that the delivery of telemedicine requires specific skills to effectively perform therapy in a remote format, and professionals require specific training in telephone communication to maintain the quality and standard of the care provided. One can debate the availability and the cost of telemedicine training sessions, however, the Ministry of Health in Oman facilitated a lot of training programs aimed at improving the competencies of mental health professionals in the delivery of telepsychotherapy services. These training initiatives are critical to ensure that professionals are not only technologically competent but are also equipped with the appropriate therapeutic skills (17). Through these training programs, the Government addressed the possible barriers that could be tackled in the implementation of telepsychology, including the familiarity of therapists with the modalities available and achieving the best quality care.

In regards to data storage for telephone-based telepsychology, all session information is documented and securely stored in the patients’ electronic medical records; in Oman, this is managed through the national electronic hospital system, which ensures confidentiality, standardized governance, and continuity of care.

The infrastructure for telemedicine in Oman is expanding, and it is progressing across other medical disciplines such as primary care, medicine, radiology, and others (22). With this development, it is valid to explore how the population perceives and accepts this method of service, particularly in telepsychology. The stigma surrounding mental health remains one of the factors affecting the acceptability in the first place. Findings from this study showed that telepsychology could serve as an effective approach to address stigma-related issues, making clients more comfortable to engage with less reluctance, which is often associated with face-to-face therapy. Findings from a study conducted in 2022 (17) showed how strongly cultural norms can impact both the clients’ and therapists’ experiences of telephone-based psychotherapy. In Oman, discussing mental health remains a sensitive topic, and having telephone therapy can be a helpful alternative. Participants from another study agreed that the experience was liberating, noting that being physically and socially distanced by a phone call gave the clients more confidence to express their issues, which might otherwise keep them to themselves or close ones (17). On the other hand, participants from our study agreed with the argument; however, they specified that tele-psychology may not be a suitable option for all mental conditions, particularly clients with active psychosis, suicidal or homicidal ideas, and emotional instability, which came in alignment with a study by Simms (23).

### Methodological considerations and limitations

Data for this study were collected by the first author, a female senior psychiatry resident. Her outsider status supported an unbiased understanding of the psychologists’ concerns, as she was unaffiliated with both the psychology department and AMH, where the participants work. This position allowed her to bring a fresh perspective, free from preconceived assumptions about the psychologists’ experiences. Moreover, AMH is the only tertiary psychiatric hospital in Oman and currently houses the country's only psychology department offering telepsychology services. Given this context, the psychologists included in the study represent the primary providers of such services nationwide, which enhances the generalizability of the findings to the Omani context.

This study has several limitations. Firstly, it focused solely on psychologists’ perceptions; future research could incorporate clients’ perspectives to provide a more comprehensive view of telepsychology. Additionally, insights from policymakers and information technology experts may further influence the development and implementation of telepsychology services. Future studies could expand upon the current findings to help build a foundation for more comprehensive telepsychology services across various levels of care. Moreover, only the first three domains of the GDEISST framework were assessed; these were chosen because they are most directly related to patient-centered care and user experience, which are critical for understanding the feasibility and effectiveness of telephone-based telepsychology from the psychologists’ perspective. The remaining two domains of the GDEISST framework could be explored in future research by including additional stakeholders such as policymakers and IT professionals.

Given the significance of integrating technologies such as telephone-based psychotherapy, results from this study support the sustainable implementation of the services with improvements in accessibility, quality, and satisfaction from both providers and clients, as it is a valuable tool for increased access to mental health services. Continued research is needed on the insights or practices of telephone-based psychotherapy among psychologists from different centres in the private sector, across the regions of Oman.

## Data Availability

The raw data supporting the conclusions of this article will be made available by the authors, without undue reservation.
